# Fulminant Disseminated Coccidioidomycosis With Histoplasma Antigen Cross-Reactivity

**DOI:** 10.7759/cureus.58129

**Published:** 2024-04-12

**Authors:** Charles Lanks

**Affiliations:** 1 Pulmonary and Critical Care Medicine, Harbor University of California, Los Angeles Medical Center, Torrance, USA; 2 Respiratory Medicine, Lundquist Institute for Biomedical Innovation, Torrance, USA

**Keywords:** histoplasmosis, miliary, cross-reactivity, histoplasma, disseminated, fulminant, coccidioidomycosis, coccidioides

## Abstract

A 79-year-old man with type II diabetes mellitus and recently diagnosed idiopathic thrombocytopenic purpura presented to the Emergency Department with progressive dyspnea over the course of two weeks. He was found to have diffuse miliary nodules, dense cavitary consolidation, and widespread cystic changes on chest imaging and died within 48 hours of admission to the hospital. His serum Coccidioides antibody and urine Histoplasma antigen were both positive. He later grew *Coccidioides immitis* from the blood, supporting the theory that Histoplasma positivity was likely the result of antigen test cross-reactivity. Coccidioidomycosis typically presents with mild, self-limited symptoms, but may also disseminate rapidly, causing fulminant, life-threatening disease. Prompt recognition of risk factors for fulminant coccidioidomycosis and understanding flaws in serologic testing are essential to the appropriate diagnosis and management of this disease.

## Introduction

Exposure to Coccidioides spp. results in clinically significant infection in approximately one-third of cases [1,2]. Of these infections, most are self-limited and resolve without treatment [3]. However, more severe diseases with extrapulmonary involvement can occur in <1% of cases [4]. Here we present a case of fulminant, disseminated coccidioidomycosis in a patient recently started on oral corticosteroids. He rapidly progressed to respiratory failure and shock and passed away within 48 hours of presentation to the hospital. The diagnosis of coccidioidomycosis was suggested on the basis of serologic testing but confounded by a positive urine antigen test for histoplasmosis. This was likely the result of Histoplasma antigen's cross-reactivity with Coccidioides, a theory that was later supported by fungal blood cultures posthumously growing *Coccidioides immitis* after four weeks.

## Case presentation

A 79-year-old man with type II diabetes mellitus and a recent diagnosis of idiopathic thrombocytopenic purpura (ITP) presented to the Emergency Department with progressive shortness of breath over the course of two weeks. He had recently been admitted at a nearby hospital for three weeks where he was diagnosed with ITP and started on prednisone at an unknown dose. His dyspnea was associated with a non-productive cough, mild chest pain, and intermittent chills, especially at night. He reported intentional weight loss amounting to approximately 20 pounds over the course of the last month as a result of diet change. He was originally born in Mexico and had recently traveled there with his family, returning to his current place of residence in the southwestern United States about one month ago, just prior to his recent hospitalization.

His initial examination was significant for a heart rate of 110 beats per minute, respiratory rate of 30 breaths per minute, and oxygen saturation of 80% while breathing ambient air. He was afebrile with a blood pressure of 106/61 at the time of presentation. His physical exam revealed diffuse inspiratory crackles in all lung fields but was otherwise unremarkable without neurologic, skin, ocular, joint, or musculoskeletal findings. Initial complete blood count (CBC) was notable for a low white blood cell count of 3.4x10^3^ cells/µL and a low platelet count of 93x10^3^ cells/µL. The metabolic panel was unrevealing but his hepatic panel revealed elevated alkaline phosphatase to 278 U/L, aspartate aminotransferase (AST) of 209 U/L, alanine transaminase (ALT) of 164 U/L, and total bilirubin of 2.6 mg/dL. Lactate was elevated to 4.0 mmol/L.

**Figure 1:**
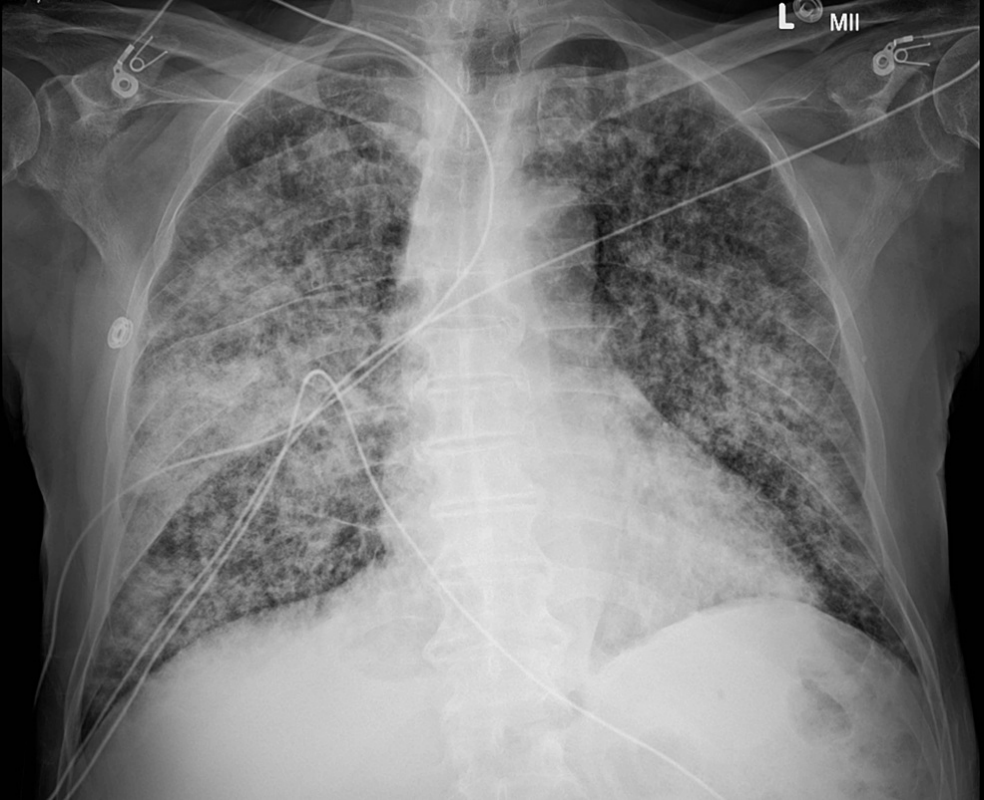
Portable semi-upright anteroposterior chest X-ray demonstrating a diffuse reticulonodular pattern with denser consolidation in the right mid-lung field.

**Figure 2:**
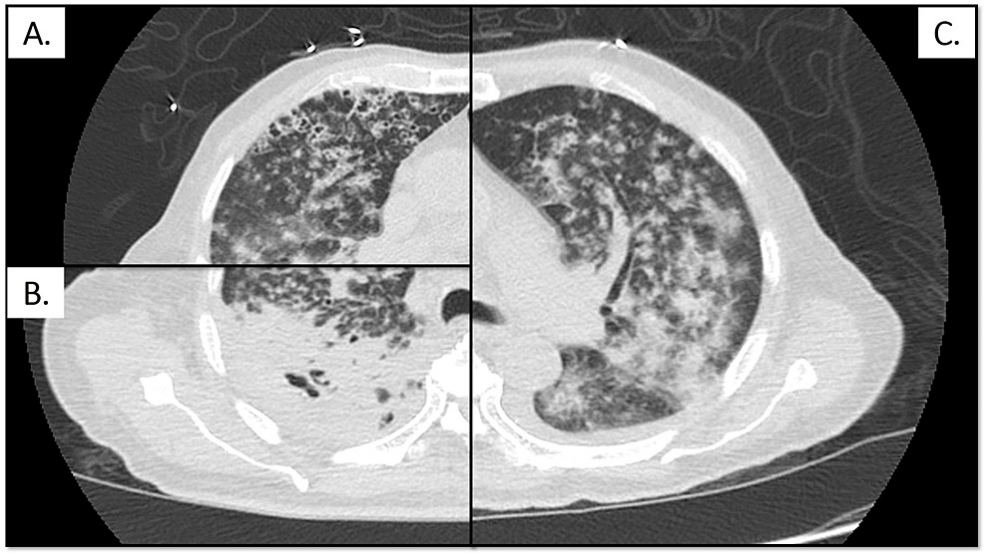
Representative sections from axial CT of the thorax showing A) thin-walled cystic changes, B) cavitary consolidation, and C) diffuse miliary nodules with surrounding ground glass in different areas of the lung, all of which can be seen in disseminated coccidioidomycosis.

His initial chest X-ray was noted to have diffuse airspace opacities and consolidations (Figure 1) and was followed up with CT of the thorax, which revealed diffuse bilateral nodular infiltrates in a miliary pattern, bronchiectasis, widespread cystic changes, and dense cavitary consolidation in the right lung (Figure 2). Shortly after the presentation, he decompensated rapidly from both a respiratory and hemodynamic perspective and required mechanical ventilation and vasopressor support. In addition to fluid resuscitation, he received empiric broad-spectrum antibiotic coverage with vancomycin, meropenem, doxycycline, and voriconazole as well as stress dose corticosteroids in the setting of refractory shock.

No bacteria were identified by Gram stain or bacterial culture from blood, sputum, or urine. No acid-fast bacilli were identified in the blood, sputum, or urine. Based on the patient’s previous debilitating hospitalization, advanced age, previous discussions with his family regarding goals of care and overwhelming current illness, the decision was made by the family to transition to comfort-focused care, and he passed away within two days of his presentation to the hospital. His serum Coccidioides antibodies were positive by immunodiffusion as were his urine Histoplasma antigen (0.69 ng/mL) and serum (1-3)-β-d glucan (>500 pg/mL). HIV testing was negative. Four weeks posthumously, his fungal blood cultures grew mold, which was identified as* Coccidioides immitis* by a DNA probe.

## Discussion

Coccidioidomycosis is an infection most commonly caused by *Coccidioides immitis*, a fungal pathogen that is endemic to the southwestern United States and Latin America [1,2]. More recently, infections are less clearly confined to a particular geographic region, possibly as a result of climate change. [5] The majority of exposures (>60%) do not result in clinically apparent infection [1,2,3]. More severe diseases are thought to be the result of larger inoculum size [3] (hence the occurrence of environmental outbreaks such as during earthquakes) and host factors such as immunocompromise [6]. Exposure typically results from inhaled airborne arthroconidia in the environment that has emerged from ruptured spherules during the parasitic phase of the organism’s life cycle. Human-to-human transmission does not occur. Some 20,000 cases are reported annually in the United States with rates of infection on the rise, but fulminant disease occurs in <1% of all cases [4].

When significant infection does occur, patients typically have symptoms of lower respiratory tract infection including fever, cough, and sputum production [1]. These symptoms often resolve spontaneously without antifungal therapy [1,2]. In fulminant disseminated disease, however, patients present with an advanced or accelerated course including involvement of extrapulmonary sites such as the central nervous system, skin, joints, and bone [4]. Our patient presented with nearly every radiographic lung abnormality that can be seen in pulmonary coccidioidomycosis, including consolidation, cavitation, cysts, bronchiectasis, and diffuse miliary nodules, the last being associated with hematogenous dissemination [7]. Some of these changes, such as bronchiectasis, were suggestive of pre-existing chronic lung disease or a more chronic infectious course, but in the setting of recently initiated corticosteroids for ITP, disease progression likely accelerated in the days prior to presentation. Diabetes mellitus may have also been a contributor [4].

Although our patient’s residence in the southwestern United States, recent travel to Mexico, and clinical course were all suggestive of fulminant coccidioidomycosis, the clinical picture was briefly confused by a positive Histoplasma urine antigen. The gold standard for the diagnosis of coccidioidomycosis remains either growth and identification in culture or by direct histopathologic identification of spherules in infected tissue [1,2,3]. In cases of fulminant disease, cultures may take too long to result and patients may be too unstable for biopsy of internal sites, particularly the lungs. In such cases, we often rely on clinical pre-test probability and serologic examinations to inform our diagnosis. In our patient, Coccidioides antibodies were detected in the serum by immunodiffusion. True disseminated infection was later confirmed through the identification of *Coccidioides immitis* in fungal blood cultures. The positive Histoplasma urine antigen was likely a false positive result due to antigen cross-reactivity. Cross-reactivity between Coccidioides and Histoplasma antigens is generally estimated at approximately 20% [8], although in one case series, positive Histoplasma antigen was detected in 79% of patients with confirmed coccidioidomycosis. [9] Conversely, Histoplasma cross-reactivity may also be observed in Coccidioides antibody testing. [9] Therefore, pre-test probability derived from clinical context is of paramount importance in fulminant disease. In our patient, the pre-test probability for both coccidioidomycosis and histoplasmosis was high. In the absence of an autopsy, this was only clarified after positive blood cultures.

Outcomes associated with disseminated coccidioidomycosis, especially with fulminant presentations such as patients with septic shock, are extremely poor with mortality rates >70%. [4] Mortality is likely related to a combination of delayed diagnosis and undertreatment with antifungal therapy. It is also likely that our patient was severely debilitated at baseline following his recent hospitalization, further contributing to his rapid deterioration. Liposomal amphotericin B is recommended for empiric therapy in cases of fulminant disease or azole therapy as a second line. [4]

## Conclusions

Fulminant disseminated coccidioidomycosis is a rare disease that is associated with high mortality. Effective treatment depends on prompt recognition and timely initiation of antifungal therapy. While diagnosis is often made on the basis of serologic testing, these studies are not without inherent flaws. Cross-reactivity between Coccidioides and Histoplasma serologies has the potential to confuse clinical decision making and results should always be considered in the context of pre-test probability of infection.
